# MyTrack+: Human-centered design of an mHealth app to support long-term weight loss maintenance

**DOI:** 10.3389/fdgth.2024.1334058

**Published:** 2024-04-22

**Authors:** Yu-Peng Chen, Julia Woodward, Meena N. Shankar, Dinank Bista, Umelo Ugwoaba, Andrea Brockmann, Kathryn M. Ross, Jaime Ruiz, Lisa Anthony

**Affiliations:** ^1^ Department of Computer and Information Science and Engineering, University of Florida, Gainesville, FL, United States; ^2^ Department of Computer Science and Engineering, University of South Florida, Tampa, FL, United States; ^3^ Department of Clinical and Health Psychology, University of Florida, Gainesville, FL, United States

**Keywords:** mHealth apps, human-centered design, weight management, adaptive interventions, self-monitoring, feedback, visualization, behavior change

## Abstract

A growing body of research has focused on the utility of adaptive intervention models for promoting long-term weight loss maintenance; however, evaluation of these interventions often requires customized smartphone applications. Building such an app from scratch can be resource-intensive. To support a novel clinical trial of an adaptive intervention for weight loss maintenance, we developed a companion app, MyTrack+, to pair with a main commercial app, FatSecret (FS), leveraging a user-centered design process for rapid prototyping and reducing software engineering efforts. MyTrack+ seamlessly integrates data from FS and the BodyTrace smart scale, enabling participants to log and self-monitor their health data, while also incorporating customized questionnaires and timestamps to enhance data collection for the trial. We iteratively refined the app by first developing initial mockups and incorporating feedback from a usability study with 17 university students. We further improved the app based on an in-the-wild pilot study with 33 participants in the target population, emphasizing acceptance, simplicity, customization options, and dual app usage. Our work highlights the potential of using an iterative human-centered design process to build a companion app that complements a commercial app for rapid prototyping, reducing costs, and enabling efficient research progress.

## 1 Introduction

In recent years, there has been a growing interest in utilizing mobile health (mHealth) applications (apps) to support and enhance various aspects of healthcare (1). Obesity remains a substantial public health challenge in the United States (2), and extended care programs have proven effective in supporting long-term weight loss maintenance (3, 4). In the weight management arena, researchers have been using smartphone apps that allow individuals to track weight and weight-related behaviors (e.g., dietary intake and physical activity) to investigate novel adaptive intervention models, such that intervention may be “triggered” by different patterns in individual behavior (5–7). To provide extended-care support at times when individuals are at high risk for weight regain, our team has designed and developed an adaptive weight maintenance intervention (8). The evaluation of such an intervention often requires development of an instrumented app with customized functionalities. However, building an app from scratch can be resource-intensive, especially when the main focus is on intervention development and outcomes assessment rather than comprehensive app development.

An alternative approach is to develop a companion app that harnesses the capabilities of the main commercial app through its open-source API. The commercially popular weight management app FatSecret (9) offers an API (10), logging interfaces, and comprehensive databases for tracking weight-related data; however, further instrumenting was needed to meet our specific goals, such as sending customized questionnaires. Thus, to support the implementation of our intervention and its evaluation in a randomized controlled clinical trial (8), we designed and developed a companion app, which we called the MyTrack+ app, to pair with the FatSecret app. Our main goal was to create a seamless logging experience for participants, ensuring smooth data collection. MyTrack+ integrates data from FatSecret and a BodyTrace smart scale (11), enabling participants to log and self-monitor their health data, while also incorporating customized questionnaires and timestamps to support our research aims. These ecological momentary assessment (12) questionnaires allow us to collect data necessary for implementing our intervention and enabling future exploratory studies. As a supplementary objective, we also integrated evidence-based behavior change techniques aimed at increasing participants’ motivation, self-efficacy, and app engagement, thereby enhancing adherence to their health objectives.

To ensure the effectiveness and user-friendliness of MyTrack+, we followed an iterative, user-centered approach. Initially, we developed app mockups and created a high-fidelity prototype based on expert evaluations. Subsequently, we conducted a usability study with 17 university students and refined our app based on user feedback and insights from our health experts. Finally, we conducted an in-the-wild pilot study with 33 participants from the target population: adults from the general public who had reported recent weight loss and who were interested in weight loss maintenance. This pilot study provided valuable findings related to acceptability and usability, and we further refined our app based on these findings. This approach facilitated rapid prototyping, iterative testing, and refinement of the app, while incorporating user feedback and the expertise of health experts in weight loss maintenance. Currently, our app has been deployed in an ongoing clinical trial evaluating an adaptive intervention for supporting long-term weight loss maintenance (8).

Our work contributes to the literature by presenting an example of how user-centered design can benefit mHealth research. We document the iterative design process of a tailored mHealth app, specifically designed for research purposes, and its seamless integration with a commercial app. We discuss the implications of our design process, highlighting the potential benefits of leveraging commercial apps for rapid prototyping, thereby reducing implementation costs and enabling researchers to make efficient progress in their investigations for similar projects.

## 2 Related work

We present relevant prior work in the areas of (1) the design and use of mHealth apps for health behavior change, and (2) the use of user-centered design processes in mHealth apps in general.

### 2.1 mHealth apps for health behavior change

Smartphone app-based interventions have gained significant attention in recent years (1). Researchers (13, 14) have identified the benefit of various behavior change techniques, such as goal setting, self-monitoring, feedback, and social support, for improving dietary intake and physical activity within app-based interventions. Zhao et al. (13) conducted a literature review of 23 articles that focused on the use of mobile phone apps to promote health behavior changes in peer-reviewed journals. The authors found that out of the reviewed studies, 17 of them reported statistically significant outcomes indicating a positive influence on the desired behavior change. Notably, self-monitoring emerged as the most frequently utilized behavior change technique, being employed in 12 of the studies. Dounavi and Tsoumani (14) also conducted a systematic literature review with the specific goal of identifying the existing evidence on the effectiveness of mobile health technology in promoting weight management behaviors, such as physical activity and healthy eating. Out of the 39 analyzed studies, the authors found that high levels of engagement with a mobile health app led to satisfactory treatment adherence, resulting in successful weight loss and maintenance.

Nevertheless, there are systematic literature reviews that find only modest evidence in support of the effectiveness of mobile apps in improving health behaviors or outcomes (15–17), in contrast to the positive evidence mentioned earlier. Based on a review of 27 studies, Schoeppe et al. (15) stated that multi-component interventions seem to be more effective than standalone app interventions and emphasized the need for further confirmation through controlled trials. Furthermore, current mHealth apps often provide extensive functionalities and complex interfaces, which may not necessarily contribute to their effectiveness (18–20). Lyzwinski et al. (18) conducted a literature review of qualitative studies focused on user perspectives and experiences with mHealth for weight loss. From their review of 20 articles, the authors identified that the most preferred apps were those that were simple and easy to use. Haggag et al. (19) performed an extensive analysis of mHealth app user reviews by extracting and translating over 5 million user reviews for 278 mHealth apps. The authors’ findings revealed that providing users with more information or functionalities than necessary can result in user frustration and reduced app usage.

This background presents an opportunity for developing a minimalist app that only focuses on key factors for behavioral change, which we emphasize in our work. Together with trained interventionists and behavioral health experts, we are using our app in an ongoing clinical trial to assess adaptive interventions aimed at supporting long-term weight loss maintenance.

### 2.2 User-centered design for mHealth apps

Previous research has emphasized the importance of *user-centered design*^1^ processes in developing effective mHealth apps for various health domains, such as self-management of chronic conditions (21, 22), mental health (23), persons living with HIV (24, 25), women in substance use recovery (26), and participants with fall risk (27). Schnall et al. (24) conducted formative research, which included focus groups, participatory design sessions, and usability evaluations, to guide the development of a health management app for individuals living with HIV. Their review of 15 existing apps meeting their inclusion criteria revealed that none of them integrated all the functionalities identified during their formative work, pointing to a significant lack of clinically-backed design choices in current mHealth apps. The authors further utilized a user-centered model to iteratively develop and refine mock-ups, creating an mHealth app tailored for persons living with HIV (25). Their findings demonstrated that a user-centered approach offered a deeper understanding of their target users’ specific requirements and facilitated the creation of an mHealth app that better aligned with user needs. Additionally, Eaves et al. (26) applied user-centered design in developing an mHealth app to support women in substance use recovery. Through an iterative design process, the authors showed that users’ feedback helped tailor an mHealth app to maximize usability, access, and safety for this at-risk population. Lastly, Hsieh et al. (27) aimed to provide personalized fall risk screening for clinical populations, including older adults, individuals with Multiple Sclerosis, and wheeled-device users, by utilizing mHealth apps. The authors developed the interface of each app with a user-centered design approach through iterative usability testing and semi-structured interviews. The authors then tested their apps in real-world settings and demonstrated the effectiveness of their apps in measuring fall risk (comparable to clinical assessments) to enhance user safety.

To develop our minimalist weight management app, we employed a user-centered iterative design process to understand users’ mental models, focusing solely on essential features. This approach enabled efficient progress in our research investigation and simpler data logging to support our research goals.

## 3 Designing MyTrack+: goals and features

We present the initial set of design goals and app features, brainstormed in concert with our behavioral health expert team members to ensure the MyTrack+ app would meet all the goals of the broader clinical trial.

### 3.1 Design goals

Our main goal was to support the implementation and evaluation of an adaptive weight maintenance intervention. Prior work had established that key data points required for such an intervention include participants’ self-weighing frequency, self-monitored dietary intake and physical activity, and self-rated measures of weight-related variables (e.g., hunger) (28). Our clinical trial (8) serves as a testbed for an automated “trigger” algorithm which can detect when participants may be at risk of relapse based on their logging or other lapses (29). Thus, our overarching goal was to facilitate data collection during the clinical trial by creating a seamless and effortless logging experience for the participants. Additionally, we aimed to employ behavior change techniques to support participants in achieving their health objectives. By integrating evidence-based strategies, we aimed to enhance motivation, engagement, and self-efficacy, supporting sustainable behavior change and promoting successful long-term weight loss maintenance. Informed by guidance provided by health experts in long-term weight maintenance and prior work on applying behavior change techniques for health behavior change (30), we formulated the following design goals for our study.

#### 3.1.1 Goal 1 (G1): enhance usability for effortless logging

We aimed to minimize the effort required for participants to log their data by designing an intuitive and user-friendly interface that is easily accessible and navigable. By reducing the effort needed to log data, participants are more likely to engage with the app consistently (14). When the interface is intuitive and user-friendly, it enhances the overall user experience and encourages active participation (14).

#### 3.1.2 Goal 2 (G2): deliver necessary instruments

We aimed to deliver research-oriented questionnaires to collect data necessary for implementing the adaptive weight intervention and enabling further exploration. The ubiquitous nature of mobile devices enables low-effort self-reporting in real-time through ecological momentary assessment (EMA) (31). In mHealth apps, EMA measures (e.g., questionnaires assessing an individual’s thoughts, feelings, and behaviors and the context in which these occur) are usually delivered repeatedly over time, in the natural environment (31). In our case, questionnaires prompt participants to self-rate, on 7-point Likert scales, factors that had been hypothesized previously to be associated with weight regain (28).

#### 3.1.3 Goal 3 (G3): facilitate self-monitoring and provide feedback

In addition to collecting essential research data, the previous two goals serve the purpose of facilitating self-monitoring and self-reflection. We further aimed to deliver personalized health information to participants in the form of summary graphs, providing tailored feedback on their progress. According to Bandura’s social cognitive theory (SCT) (30), self-monitoring increases the user’s awareness of their progress and feedback provides an opportunity for them to adjust their strategy (30). Self-monitoring refers to a person’s action of keeping a record of details related to performance of the target behavior (e.g., logging the duration and intensity of performing certain physical activities). Such action is a part of the self-regulatory mechanism that is required for beneficial behaviors to be achieved and maintained (30). Moreover, for goals to be effective, summary feedback on self-reported details is also crucial. Such feedback provides an opportunity for individuals to adjust the level or direction of their effort or to adjust their strategies to match what the goal requires (30). Prior work showed that a system that facilitates the user’s self-monitoring and provides feedback can effectively increase physical activity (32) and motivate healthy eating (33).

#### 3.1.4 Goal 4 (G4): increase motivation and self-efficacy

Our secondary objective was to harness the power of behavior change techniques inspired by the principles of goal-setting theory to increase participants’ motivation and self-efficacy. Locke and Latham’s goal-setting theory (GST) (34) states that having a goal is a crucial cognitive determinant of human behavior and performance. According to the GST (34), there is a positive, linear relationship between goal difficulty and levels of effort and performance. The authors found that effective goals should be challenging enough to induce effort for an individual to be motivated but should not be so difficult that they cause repeated failures. Goal setting has been shown to be an important factor in supporting behavior change in various health fields (35, 36). Furthermore, Bandura’s social cognitive theory (SCT) (30) holds that self-efficacy is a major determining factor for behavior change. Self-efficacy is an individual’s belief in their ability to execute a certain behavior in a given situation. Repeated successes play a significant role in boosting self-efficacy, as past achievements have a notable influence on self-perception (30). Therefore, the keys to accomplishing long-term health behavior changes involve setting intermediate goals and making persistent efforts towards their achievement. Individuals can enhance their self-efficacy by actively monitoring their own behavior and receiving feedback that highlights progress towards goal attainment (34). Previous studies have focused on increasing the user’s self-efficacy to motivate health behavior change (37, 38).

#### 3.1.5 Goal 5 (G5): promote app engagement and adherence to health objectives

To achieve this goal, rooted in behavior change theories (30, 39), we aimed to encourage app engagement and adherence to health objectives by incorporating notifications and expert support into our system. The Fogg Behavior Model (39) emphasizes the importance of providing triggers to increase app engagement and adherence. Prior work has also pointed out that mHealth app notifications can aid in behavioral change through increasing user app engagement and adherence to health objectives (40). Additionally, the Supportive Accountability model proposed by Mohr et al. (41) argues that human support increases adherence to electronic health interventions through accountability to a human coach. Social persuasion provided by a human coach can also enhance an individual’s self-efficacy, thereby increasing motivation (30). Being encouraged to perform certain behavior by others through suggestions can enhance an individual’s belief in their capability of successfully executing such behavior (42). Previous studies found that users consider consultation and communication with health experts to be a crucial functionality in mHealth apps (24, 43).

### 3.2 Initial design features

To achieve our design goals, we considered the following initial design features in MyTrack+.

#### 3.2.1 Interfaces for logging weight-related behaviors

While this section primarily focuses on designing MyTrack+, to achieve **G1** and also streamline the software engineering process, we utilized the existing features in a well-established commercial app, FatSecret, and a BodyTrace smart scale (Table 1). FatSecret provides an interface for logging consumed food and drinks, including portion sizes and nutritional information such as calories and macronutrient/micronutrient composition. It also includes features that enhance its usability, such as barcode scanning for food items and the “Recently Eaten” and “Most Eaten” options, reducing logging efforts. Users can also log exercises with duration and access a comprehensive database of physical activities. Additionally, our system incorporates the use of a BodyTrace smart scale that utilizes cellular network connectivity to transmit participant weight data to BodyTrace servers. This eliminates the need for participants to manually log their weight data, as developers can directly request the information from the servers.

**Table 1:** Our mHealth system incorporates three main components: (1) our MyTrack+ app, (2) the FatSecret commercial app, and (3) a BodyTrace smart scale.

Application/Device	Data Type	Features
MyTrack+ app	Questionnaire	Summary graphs, Notifications, Goal setting, Health expert support
FatSecret app	Dietary intake, Physical activity	Self-logging user interface, Databases of nutritional and exercise information
BodyTrace smart scale	Weight	Automatic weight logging

This system was designed to facilitate the self-monitoring of health data relevant to weight management, including weight, dietary intake, physical activity, and weight-related factors (via questionnaires).

#### 3.2.2 Navigation

Achieving effortless logging (**G1**) requires an intuitive app with easy navigation. We focused on facilitating both cross-app navigation (from MyTrack+ to FatSecret) and within-app navigation (between different pages within our app).

#### 3.2.3 Research-oriented questionnaires

To achieve **G2**, we utilized MyTrack+ to conduct EMA through weekly and end-of-week check-in questionnaires. Questionnaire content was developed by our team of health experts, while the software engineering team dedicated their efforts to designing the user interface. This feature also supports the achievement of **G3** by allowing users to assess their progress through rating various factors that were previously hypothesized to be linked to weight regain (28).

#### 3.2.4 Notifications

To achieve **G2**, we implemented notifications that appear both outside the app (i.e., push notifications) and inside the app (i.e., in-app notifications). These notifications serve as reminders for users to reflect on their weekly progress while also accomplishing **G5**. A survey found that designers use notifications as a way to facilitate behavioral change by increasing user engagement with the app and promoting adherence to health objectives (44).

#### 3.2.5 Overall summary graphs

To achieve **G3**, we included summaries for calories remaining, physical activity, and weight in the form of summary graphs, as providing visual feedback on self-reported data is an effective strategy for boosting physical activity levels and promoting healthier eating habits (14). These graphs also aid in accomplishing **G4**, as they enhance self-efficacy by empowering the user to visualize and review their past achievements.

#### 3.2.6 Goal setting and monitoring

To achieve **G4**, we implemented features that enable users to set their goals. We also implemented features that enable the user to track their progress towards intermediate sub-goals, contributing to both **G3** in providing feedback on self-reported data and **G4** by promoting self-efficacy through the accomplishment of repeated small successes.

#### 3.2.7 Expert support

To achieve **G5**, we created a support page that facilitates communication between users and health experts, as accountability to a human coach has been demonstrated to enhance adherence (41). This feature also contributes to **G4** by promoting self-efficacy through social encouragement (30).

## 4 Iterative design of MyTrack+

**Figure 1:**
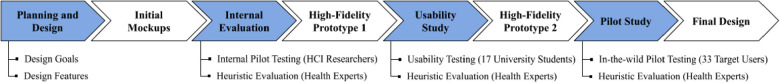
Our user-centered design process involving iterative evaluation and incremental refinement of app prototypes. The elements colored in blue indicate stages where we conducted brainstorming sessions, evaluations, and/or studies. Specifically, (1) HCI researchers were recruited for internal evaluation, (2) Computer Science students were recruited for the usability testing, and (3) the target users were recruited for the pilot study. We engaged our health experts for each of these stages to ensure that the incremental changes we made were aligned with recommendations from the weight management literature. The elements colored in white represent the result of the previous element.

To achieve our design goals and determine how to support the features we brainstormed, we followed a user-centered approach with iterative evaluation of prototypes and corresponding incremental changes, encompassing multiple stages (Figure 1). The process involved first developing app mockups and designing the backend system. We developed the first version of a high-fidelity prototype based on feedback from health experts in our team and through internal pilot tests with our human-computer interaction (HCI) researchers. Subsequently, we conducted a usability study with 17 university students to gain insights into ease of use, preferences, and technical issues. We refined our app based on feedback received from users and insights provided by our health experts. Finally, we conducted an in-the-wild pilot study involving 33 participants from the general public, and then further refined our app based on feedback from both participants and our health experts.

Our transdisciplinary team included faculty, staff, and graduate students with expertise in obesity treatment, clinical psychology, computer science, and human-computer interaction (HCI). The four-member health expert team comprised of a faculty clinical psychologist with 17 years of experience in obesity treatment, a registered dietitian with 21 years of research experience, and two Clinical and Health Psychology graduate students with training in obesity treatment. The six-member computer science team comprised of two faculty members with a combined total of 25 years of research experience in HCI and 15 years of industrial experience in software engineering, and four graduate students with training in computer science and HCI.

### 4.1 Initial mockups and backend design

**Figure 2:**
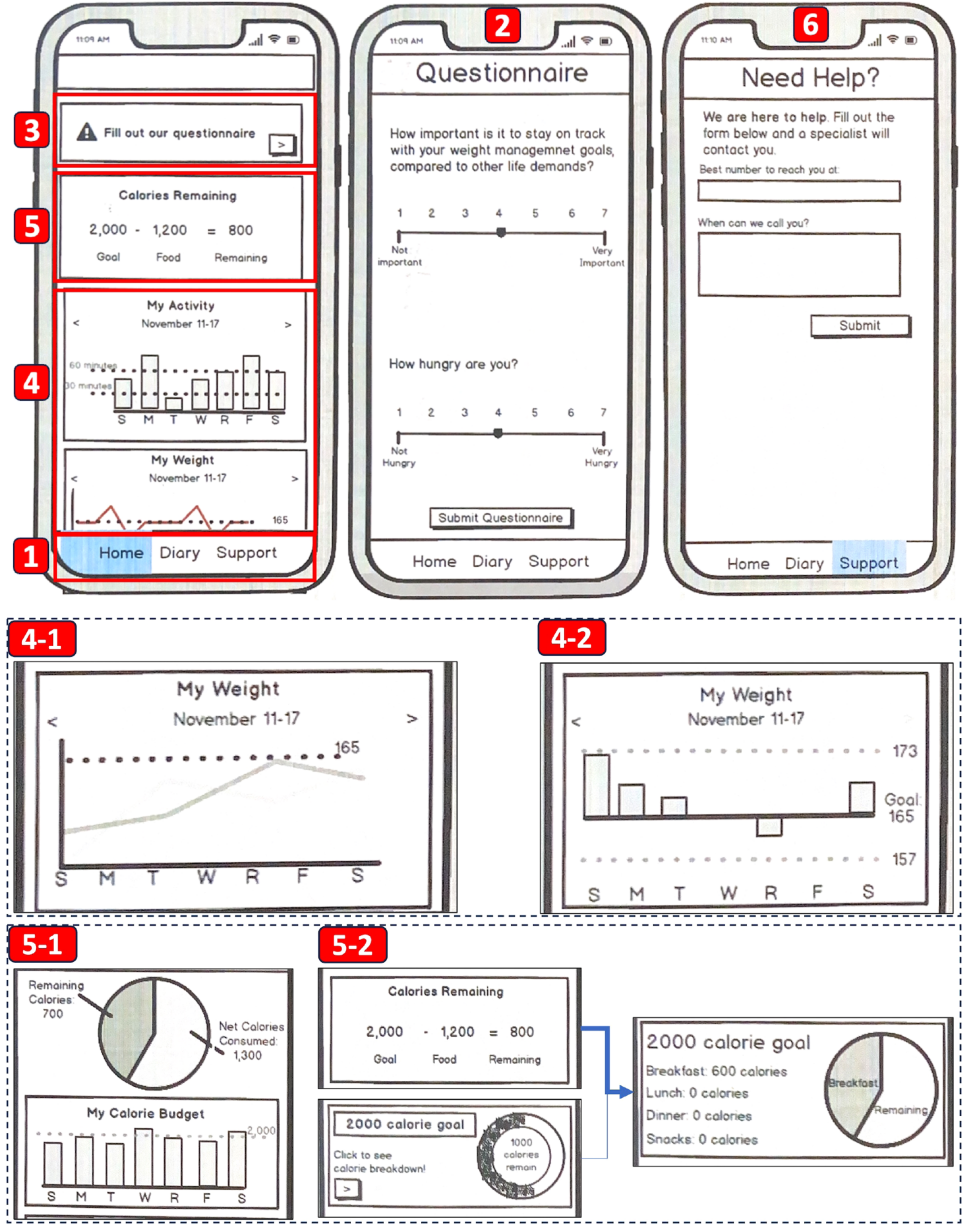
The app mockup components. The labels correspond to the descriptions provided in Section 4.1: (1) The navigation panel. (2) The Questionnaire screen. (3) The in-app notification. (4) The summary graphs for physical activity and weight data with two design options: (4-1) a line graph and (4-2) a bar chart. (5) The calorie intake summary with two design options: (5-1) display both daily and weekly summaries on the Home screen and (5-2) display the daily summary on the Home screen and link it to another screen for weekly summary. (6) The Support screen.

We designed the following mockup components based on our design goals and initial features (Figure 2). We provide a detailed explanation of each component in the same order as our initial design features listed in Section 3.2.

#### 4.1.1 Navigation

At the bottom of MyTrack+ (Figure 2-1), we implemented a navigation panel that includes the “Home”, “Diary”, and “Support” tabs. These tabs allow the user to navigate to the Home screen, FatSecret, and the Support screen from any screen. We implemented the “Diary” tab to facilitate the navigation from MyTrack+ to FatSecret. While we lack control over the implementation of the FatSecret app, users can navigate backward using the built-in back navigation feature on both iOS and Android devices. We also implemented arrows within in-app notifications and summary graphs to visually indicate clickable components.

#### 4.1.2 Questionnaires

**Figure 3:**
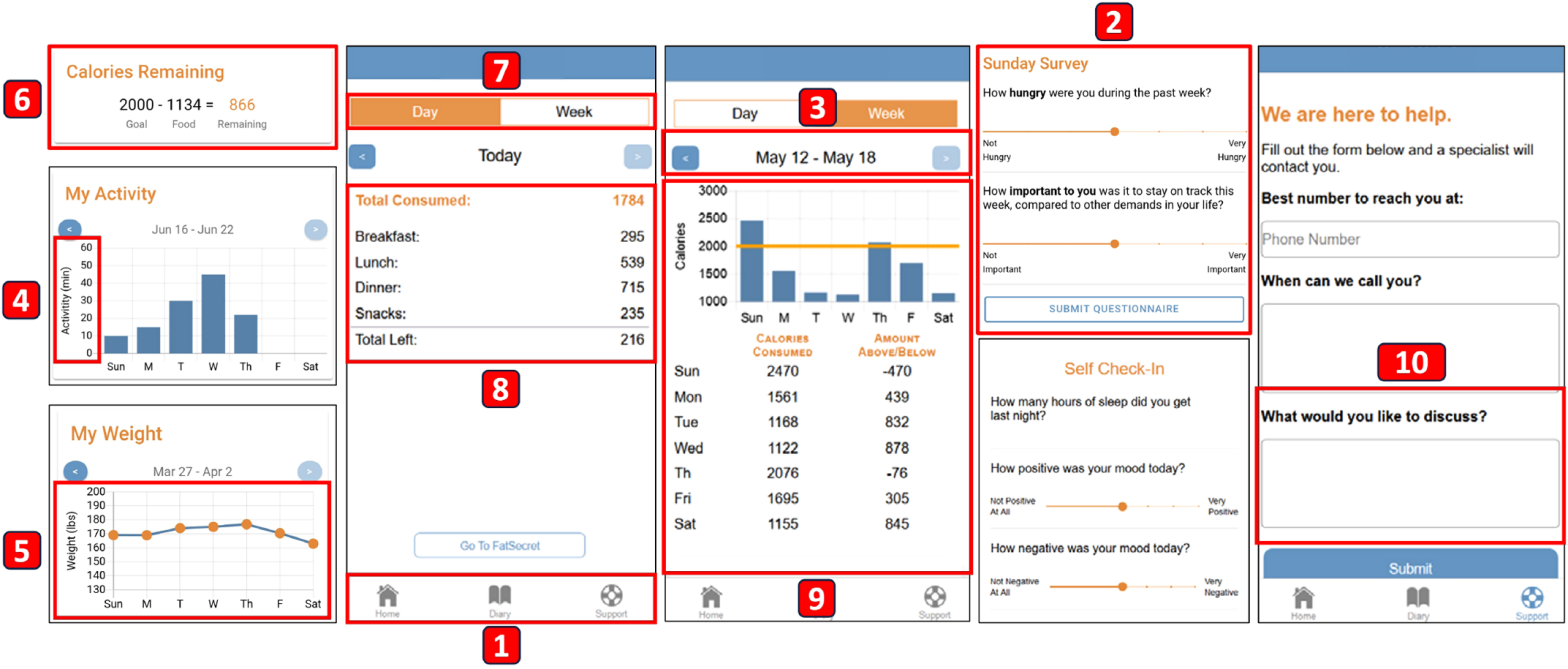
The first high-fidelity prototype. The leftmost screenshot displays the Home screen, with the components arranged from top to bottom following the same order as in the actual app. The labels’ order follows our description in Section 4.2: (1) Navigation panel with icons. (2) End-of-week questionnaire. (3) Shading of the arrows in summary graphs. (4) Physical activity summary graph. (5) Weight summary graph. (6) Daily calorie goal. (7) Tabs for selecting types of details. (8) Daily dietary intake details. (9) Weekly dietary intake details. (10) Open text input for topics on the Support screen.

We created a Questionnaire screen (Figure 2-2) that displays questions developed by our health experts. We implemented a 7-point Likert scale slider for each question, allowing users to self-rate the weight-related factors. Users can navigate to this screen through in-app notifications (Figure 2-3).

#### 4.1.3 Notifications

We designed notifications based on empirically-derived notification design recommendations in mHealth apps, including position, aesthetics, and content (44). We placed the in-app notification at the top of the Home screen (Figure 2-3) to ensure that it is prominently visible and does not interfere with the main content of the app. We also implemented a transient warning message that appears at the bottom of the user’s current screen and disappears after five seconds. This brief message can capture the attention of users who may not be actively looking at the top of the home screen or may be engaged in a different part of the app’s interface. We included an arrow to indicate that the notification is clickable and a “warning” icon to indicate the importance of clicking on the notification. To create an effective visual effect, we chose orange as the background color for the in-app notification, leveraging its complementary nature to our main color theme of gray blue.

#### 4.1.4 Summary graphs

**Figure 4:**
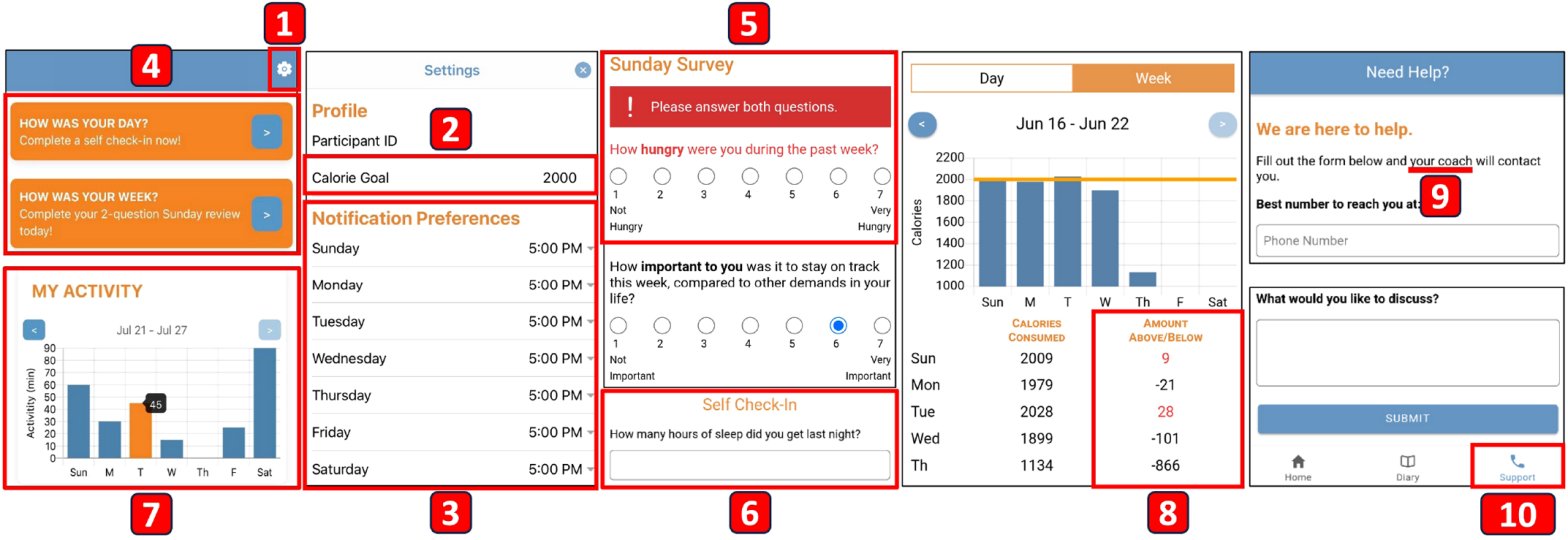
New features or changes in the second high-fidelity prototype: (1) The “gear” icon for navigating to Settings. (2) An input space where the user can set and change their calorie goal. (3) Notification preferences. (4) In-app notifications. (5) Unanswered question on the Questionnaire screen and radio buttons. (6) An open-response text box for the hours-of-sleep question. (7) Displaying numerical values when bars are clicked. (8) The values are calculated based on “consumed − goal”. (9) The term “your coach” for tailored personification. (10) The “phone” icon for the Support tab.

Our summary visualization includes graphs for physical activity and weight data (Figure 2-4). Our app displays data summary on a week-to-week basis (Sunday to Saturday), while providing the functionality for the user to monitor tracking history of previous weeks. Regarding the type of weekly summary graphs, we explored line graphs (Figure 2-4-1) and bar charts (Figure 2-4-2). Additionally, for tracking physical activity, we explored different units, including total duration in minutes or total burned calories.

#### 4.1.5 Dietary intake and goal indicators

**Figure 5:**
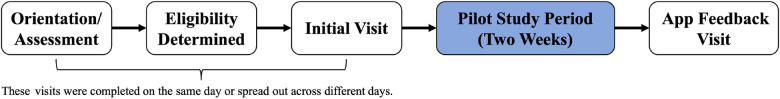
The procedure of our two-week, in-the-wild pilot study involving 33 target users.

Our app displays summary graphs for dietary intake (Figure 2-5). To display daily calorie goal and the weekly calorie intake, we considered two options: (1) display both graphs on the Home screen (Figure 2-5-1) or (2) display the daily calorie goal on the Home screen and link it to a separate screen for daily breakdown and weekly summary (Figure 2-5-2). For the daily calorie goal, we considered a balance equation, a circular progress bar, and a pie chart. For visualizing the detailed breakdown of daily and weekly calorie intake, we considered a bar chart and a pie chart, with text information displayed below and alongside. Additionally, we designed goal-monitoring features, including textual information in a daily summary indicating the calorie goal, total calories consumed, and remaining calories. In the weekly graph, a line representing the calorie goal was incorporated.

#### 4.1.6 Support screen

**Figure 6:**
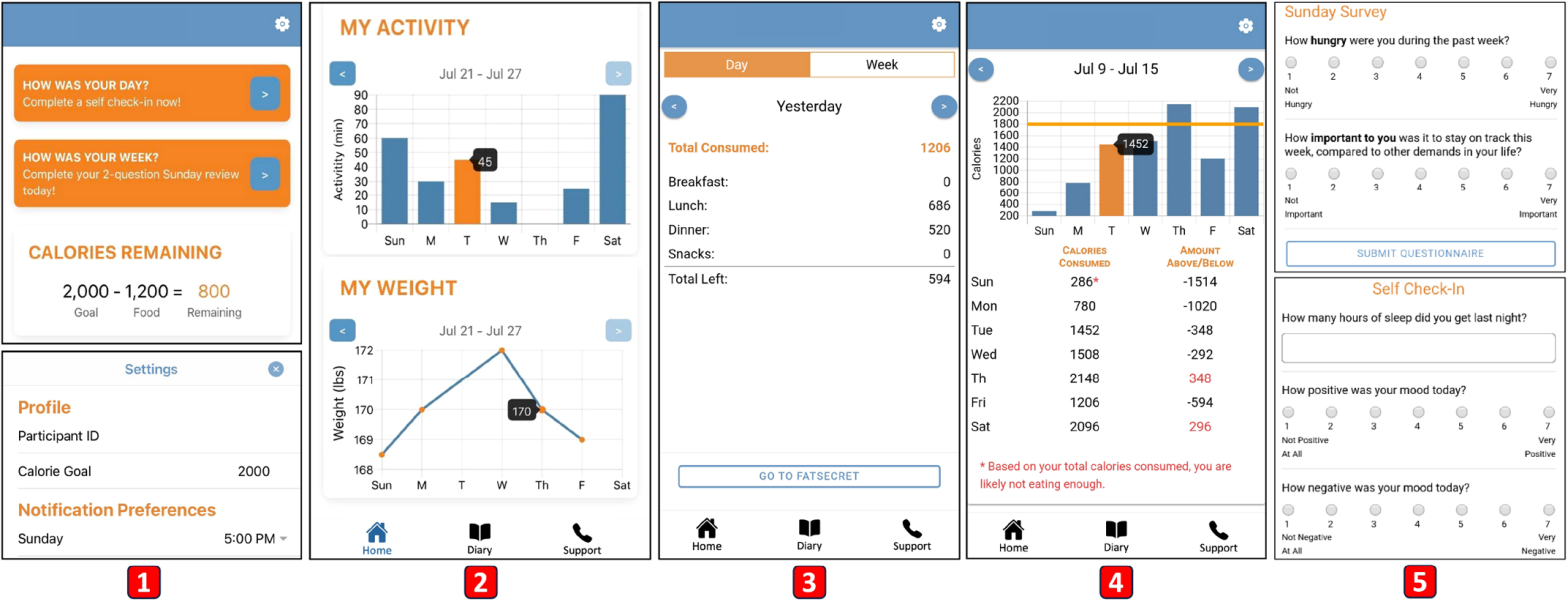
Main screens of our final design: (1) The upper part of our Home screen and the Settings screen. (2) The lower part of our Home screen with summary graphs. (3) Daily dietary intake summary. (4) Weekly dietary intake summary. (5) Questionnaires.

On the Support screen (Figure 2-6), the app presents the message: “Fill out the form below and a specialist will contact you.” We introduced a “specialist” character to indicate real human support, which has been shown to be effective in increasing engagement (40). Since our support is provided through phone, we designed open text input for the user to input their phone number and indicate their preferred call time.

#### 4.1.7 Backend design

To ensure a seamless data collection process and smooth dual app usage, the MyTrack+ app focuses on integrating data from multiple sources: FatSecret and BodyTrace. Specifically, we implemented three backend components for this feature. We integrated the Firestore Database (45) into our system for data storage. We built Google Cloud Functions (46) for synchronization between our app and FatSecret. Google Cloud runs scheduled Cloud Functions to request data from FatSecret’s server using the API they provide. We provided a “Diary” tab in the navigation panel at the bottom of the Home screen. This allows the user to navigate from MyTrack+ to FatSecret from any screen. In addition, the FatSecret API does not include timestamps for self-monitored weight, caloric intake, or physical activity. However, the inclusion of timestamps is crucial for supporting the implementation and evaluation of our adaptive weight maintenance intervention, as well as for conducting further exploratory study, such as developing novel models to proximally predict weight change and supporting future adaptive intervention development. Thus, in our backend system, we appended timestamps to the data collected in FatSecret and BodyTrace when requesting data from those applications’ servers.

### 4.2 High-fidelity prototype 1

In our iterative process of improving the app to fulfill our design goals, we first sought guidance from our team of health experts and performed internal pilot tests with our team of HCI researchers. Based on feedback from these experts and researchers, we developed the initial high-fidelity prototype (Figure 3). We detail our iterative improvement process, highlighting our design goals.

To facilitate effortless logging (**G1**) through intuitive navigation and user-friendly interfaces, we refined navigation and questionnaire components, following suggestions from our HCI researchers who drew upon previous studies in the mHealth field (14). We integrated icons into the tabs of the navigation menu, providing visual cues for easy navigation (Figure 3-1). Additionally, we highlighted the keywords within the questions in the questionnaires to draw attention (Figure 3-2), aiming for **G2**. For example, we emphasized the “hungry” keyword using bold text in the question that prompts the participant to rate their hunger: “How **hungry** were you during the past week?” Lastly, we introduced shading to the arrows in summary graphs (Figure 3-3) to indicate whether the user can navigate and review data from past weeks (**G3**).

To enhance self-awareness (**G3**), boost motivation (**G4**), and increase engagement (**G5**), we incorporated feedback from our health experts to refine components such as summary graphs, dietary intake detail, and the Support screen. For the physical activity summary graph (Figure 3-4), we used minutes instead of “calorie burned” as the unit because our intervention goals are set in minutes, based on guidelines issued by both the American College of Sports Medicine (ACSM) (47) and the Centers for Disease Control and Prevention (CDC) (48). For the weight summary graph (Figure 3-5), we selected the line graph because it effectively shows the change of weight over time. For dietary intake summary (Figure 3-6), our app displays only the daily calorie goal summary on the Home screen to avoid redundancy. We opted for a balance equation format because it was perceived as more easily understandable and preferable at a glance, while a circular progress bar received unfavorable feedback. Clicking on the balance equation redirects the user to the dietary intake detail screen, which features two tabs at the top (Figure 3-7). For daily breakdown, based on feedback from our health experts, the pie chart was deemed confusing. Thus, we opted for a vertical subtraction expression to enhance clarity (Figure 3-8). This design presents the total calories consumed for the day at the top, followed by a breakdown of calories consumed by meals (e.g., breakfast, lunch, dinner, snacks) listed below, and concludes with the remaining calories at the bottom. For the weekly dietary intake summary, we opted for a bar chart representation, as it effectively emphasizes the line indicating the calorie goal (Figure 3-9). Supplementary numerical values are included below the bar chart, displaying the calories consumed and the deviation from the goal (i.e., daily calorie goal - calorie consumed) for each day of the week. Lastly, our health experts provided feedback indicating that incorporating an open text input for users to express the topics they would like to discuss is beneficial for both the user and the health professional (Figure 3-10).

### 4.3 Usability study

To iteratively assess and enhance the high-fidelity prototype of the MyTrack+ app, we performed a lab usability study involving 17 students majoring in Computer Science from our local university. Our goal is to evaluate MyTrack+’s general usability and identify basic bugs and major usability flaws. Although Computer Science students were not our main target users, according to Nielsen (49), involving students in the domain of interest from a local university in usability testing can still yield valuable feedback. We chose to recruit students as convenience samples (50) because (1) they are easier to reach and more readily available and (2) they could also be potential users of weight management apps. We listed our study in the Computer Science department’s Research Participation System to recruit students enrolled in Computer Science classes. We excluded one participant due to missing data. The mean (SD) age of the 16 participants was 21.7 (3.3) years and 3 participants (19.8%) were women.

To address potential limitations in our lab study, which may not reflect real-world stress, and because we recruited university students who may not have a weight maintenance goal after weight loss, we used four task scenarios in the usability study to evaluate our prototype:

• **Scenario 1** (**G1**, **G3**, and **G4**). We explained the context of use for this app being to help people manage their weight after weight loss. Our first scenario asked the participant to explore the Home screen with summary information and the dietary intake detail screen with this in mind.
• **Scenario 2** (**G5**). We described a scenario where the user was having trouble staying on track with their weight management program and would like someone to contact them in order to talk about it.
• **Scenario 3** (**G2**). We described a scenario where the user was experiencing a real-world stress (e.g., promotion at work) that demanded extra hours and attention. Participants were asked to fill out the end-of-week questionnaire, which is a part of their weight management program every Sunday. The end-of-week questionnaire includes two questions prompting the participant to rate two weight-related factors, which are necessary for our adaptive intervention.
• **Scenario 4** (**G2**). We then asked the participant to fill out the weekly check-in questionnaire, under the same circumstance (i.e., experiencing a real-world stress that demanded extra hours and attention). The weekly check-in questionnaire includes 12 questions prompting the participant to rate 12 weight-related factors, which are also necessary for our adaptive intervention. In our clinical trial, this questionnaire is sent on a random day between Monday and Saturday (with participants unaware of the day that the questionnaire will be asked).

We showed the participants the MyTrack+ app and read the description of our scenarios. They were then asked to perform tasks in our app and answer questions about the usability of the app while thinking aloud (51). They then completed a System Usability Scale (SUS) questionnaire (52) and received extra course credit as compensation. Our study protocol was approved by our university’s Institutional Review Board (IRB).

### 4.4 High-fidelity prototype 2

The average SUS score was 84.53 (min = 60; max = 100; SD = 10.92), surpassing the widely accepted SUS score benchmark of 68 (53). Based on feedback from the usability study and our health experts, we further refined our app and developed the second high-fidelity prototype (Figure 4). Throughout this process, our main focus remained on achieving established design goals.

In the usability study, students noted their needs to input or change their calorie goal, mentioning that, “I was not sure how to input my target goal.” (P17) and, “It feels like you should be able to click on the section headers and get taken to a configuration screen.” (P05). Aligned with our design goal on providing goal-setting functionality to increase motivation (**G4**), we implemented a “gear” icon (Figure 4-1) on the Home screen and linked it to the Settings screen (Figure 4-2). Additionally, researchers have increasingly emphasized the importance of flexibility and customizability in mHealth apps to support user autonomy (54). Previous studies have found that customizable apps can increase users’ engagement by providing a sense of control (55). Together with feedback from our health experts, we implemented options for the user to specify their notification preferences (Figure 4-3), effectively achieving **G2** and **G5**. Based on the specified times, in-app notifications appear at the top of Home screen (Figure 4-4). The notification content was decided based on discussions among our HCI researchers and health experts, drawing from our expertise and prior work. Our goal was to deliver a motivational message (“How was your day/week?”) with a sense of urgency in responding to the questionnaire, employing an assertive tone emphasized by an exclamation mark (“Complete a self check-in now!”), following the established recommendation (40).

Clicking on the in-app notifications redirects the user to a separate Questionnaire screen. Since the questions are critical for our research purposes, our app displays a warning if there is more than one unanswered question and changes the color of the unanswered question to red (Figure 4-5). Based on feedback from the students, mentioning that, “Sometimes, when I would try to scroll, my finger would accidentally move the sliders on the responses.” (P05), which is consistent with the “fat fingers” issue for touchscreen gestures (56), we replaced the sliders with radio buttons. We also added an open-response text box to the hours-of-sleep question based on feedback from our team of health experts (Figure 4-6).

For summary graphs on the Home screen, students expressed difficulty in reading the chart, mentioning that, “If I was not paying attention I may not read the chart correctly.” (P03). Our health experts also provided similar feedback, which prompted us to implement a feature wherein numerical values are displayed when bars or points in the graphs are clicked (Figure 4-7). This feature aims to facilitate self-reflection for increasing self-awareness (**G3**). For the numerical values on the weekly dietary intake screen (Figure 4-8), we changed the direction of subtraction from “goal − consumed” to “consumed − goal” based on feedback from our health experts, stating that negative signs should be used when someone is below their goal. Interestingly, students expressed confusion about this particular change, mentioning that, “At first sight the negative values seemed a little tricky to me. Only because thinking of something in the negative might ignite a feeling of inadequate performance?” (P08). Thus, we implemented visual indicators by marking values that exceeded the calorie goal in red, aiming to strike a balance between user preferences and health expert suggestions.

Lastly, students expressed confusion about the purpose of the Support screen, stating that, “[I’m] confused if the purpose of Support is supposed to connect you with someone to encourage you to reach your goals or if it’s general support for the app overall.” (P09). Our health experts supported this feedback and recommended replacing the “support buoy” icon with a “phone” icon (Figure 4-10). We also changed the term “specialist” to “your coach” (Figure 4-9), aligning with the established recommendation of incorporating tailored personification (40).

### 4.5 Pilot study

To iteratively assess and improve the second high-fidelity prototype of the MyTrack+ app, we conducted an in-the-wild pilot study involving 33 target users from our local community who had lost at least 5% of body weight during the past two years. We excluded two participants due to missing data. The mean (SD) age of the 31 participants was 40.1 (15.6) years and 21 participants (67.7%) were women. The following are details of our two-week pilot study procedure (Figure 5).

#### 4.5.1 Recruitment

Participants were recruited using flyers, outreach to local employers/businesses, and newspaper ads; all such materials were approved by our IRB.

#### 4.5.2 Orientation and informed consent process

Participants who met initial eligibility criteria as assessed via the phone screen were invited to attend an in-person orientation visit. The orientation visit included a discussion of the pros/cons of taking part in research and specific details of the current study. Potential participants were given the opportunity to privately ask study staff any remaining questions and then, if they remained interested, were asked to provide written informed consent.

#### 4.5.3 Initial visit

Participants who met study eligibility criteria were provided a study smart scale developed by BodyTrace, Inc. (11). Study team members reviewed the MyTrack+ smartphone application and assisted participants with smartphone setup. Participants were asked to use the study smart scale and MyTrack+ application for the following two weeks before returning for a follow-up feedback visit. Participants were asked to weigh themselves each day, first thing in the morning after using the restroom but before eating/drinking, in nothing more than light indoor clothing (57).

#### 4.5.4 Pilot study period

During the two-week study period, participants were asked to (1) use study smartphone apps to track weight, caloric intake, and physical activity each day, (2) use smart scale to measure weight each day, and (3) complete questionnaires when prompted via study smartphone app. MyTrack+ pushed two questionnaires to participants each week: one 12-item questionnaire (Weekly MyTrack+ Questionnaire) delivered on a random day each week, ranging from Monday through Saturday and one 2-item end-of-week check-in (“End of Week MyTrack+ Questionnaire”).

#### 4.5.5 App feedback visit

Participants were asked to return two weeks after their initial study visit to complete SUS questionnaires and provide feedback regarding the usability/acceptability of the MyTrack+ smartphone application. Study staff measured participant weight, and completed a semi-structured interview to assess participant perception of usability and acceptability of the MyTrack+ application. Audio recordings of the semi-structured interviews were collected to document suggestions for further app development. Participants were provided with a $30 honorarium for completing this study visit.

Our study protocol was approved by our university’s IRB.

### 4.6 Final design

As the last phase of our iterative design process, we integrated the insights from the pilot study and gathered additional input from our health experts. The average SUS score of the second high-fidelity prototype was 70.48 (min = 27.5; max = 97.5; SD = 16.89), which remained higher than the typical SUS score benchmark of 68 (53). Focusing on our design goals, we continued to refine our app and created the final design of the MyTrack+ app (Figure 6), which is now in use in our ongoing clinical trial.

For the weight summary graph (Figure 6-2), our pilot participants expressed the need for more noticeable weight changes to enhance their motivation. Suggestions included providing a zoom-in functionality or reducing the unit range on the y-axis. To address the issue, we prioritized addressing the main range of weight changes while avoiding investing significant engineering effort in handling extreme edge cases. This was based on the understanding that in realistic scenarios, significant weight changes do not typically occur in a short period of time.

For dietary intake details (Figure 6-4), we included tailored feedback based on the total calories consumed, as suggested by our health experts. For example, if total calories consumed are too low below the goal, the page displays the message, “Based on your total calories consumed, you are likely not eating enough.” Notably, nearly all participants expressed concerns regarding the delay in data synchronization between MyTrack+ and FatSecret. To address this issue, we modified our Cloud Functions to request data from FatSecret’s server whenever the companion app is refreshed, effectively achieving **G1**.

## 5 Findings

We present four general themes identified throughout our design process.

### 5.1 Personalized preferences

Design preferences for an mHealth app tend to be highly personalized, varying from one user to another. Some users preferred the simplicity of MyTrack+, finding it effective in presenting relevant information clearly, stating that, “As it [MyTrack+] stands now, while there isn’t much on the homepage, it’s easy enough to navigate with a couple taps to any of the other information that someone would want to look at. So I think it’s simple enough as it is now too jumbled to put too much on the home screen.” (P418). Others, however, felt it lacked features and complexity compared to the FatSecret app, stating that, “The goal of [MyTrack+] is to show me that this is how many calories you’ve eaten during the day. It doesn’t appeal to me. FatSecret is going to show me my macro breakdown for the day.” (P427). Some users suggested adding more functionality to MyTrack+ based on their previous experiences with health-related technologies, such as providing detailed nutrition information for specific dietary restrictions and incorporating features like BMI tracking and water intake monitoring.

### 5.2 Effectiveness of behavior change techniques

Users found the process of setting a calorie goal and tracking their progress toward that goal to be highly beneficial. They appreciated visual representation of their performance, as it showed variations in calorie intake on different days, motivating them to be more mindful of their eating habits. Additionally, users emphasized the positive impact of the daily weight and weekly activity tracking features on their self-awareness. They also stated that these features empowered them to stay accountable by remembering whether they had achieved their exercise targets, leading to a positive impact on their adherence to their health goals. Furthermore, users found the in-app notification helpful in reminding them to answer questionnaires, which provided an opportunity for reflecting on health-related factors. Notably, some users extended their usage of the app and integrated it as an assistive tool in their daily lives. For example, one user made connections between their daily weight changes with their menstrual cycle.

### 5.3 User agency

Many users expressed their desire for control over how data is displayed and their need for flexibility to log for previous days. For example, when asked about the weight graph, one user mentioned that, “The increments are so minuscule that it looks like I have effectively no progress when I know I have.” (P457). They then mentioned, “I did try to see if I could adjust the increments, but I didn’t see a setting for that.” (P457).

### 5.4 Expectations in apps and connected devices

Nearly all users expressed frustration with delays in synchronization and inaccuracies in data from different sources. A common practice among many users was to immediately verify the accuracy of their recorded weight and ensure that data was correctly synced between MyTrack+ and FatSecret after self-weighing or logging data.

## 6 Discussion

We discuss the implications derived from user’s mental models and reflect on our design process.

### 6.1 Implications derived from users’ mental models

Our studies and iterative design process enabled us to not only gather information about the usability of the app and make design decisions, but also revealed insights into potential target users’ mental models of how mHealth apps like these should work. Our observations highlight the individualized nature of user preferences in mHealth app design, emphasizing the fact that there is no one-size-fits-all solution (58). This underscores the importance of adopting a user-centered design approach to understand the specific needs of the target users. We also observed that our app design has effectively enhanced users’ motivation, self-efficacy, self-awareness, app engagement, and adherence to health goals. This echoes previous work on effectively applying behavior change techniques in mHealth apps to support users’ health goals (13).

A noteworthy observation is that some participants extended the use of certain features, incorporating them as assistive tools in their daily lives. This action of adding flexibility to the use of MyTrack+ indicates the need of user autonomy for mHealth apps. In fact, researchers have emphasized the significance of user autonomy in mHealth technologies (54). Customizable apps have been shown to enhance user motivation and engagement by offering a sense of control (55). For example, systems that allow the user to manually add, edit, or delete personal health data can promote self-awareness and accountability (54). Customizable apps can also allow users to tailor app content to fit personal preferences and goals. Engaging in such customization can enhance user agency and increase the consumption of the customized content (55). As mentioned in our findings, participants expressed their desire for control over how data are displayed and their need for flexibility to log for previous days.

In the health IoT domain, interconnected smart devices form a cohesive system that empowers users to effortlessly achieve their health goals within their daily living environment. In this context, it becomes especially important to preserve human agency by helping users understand and giving them control over their AI-powered devices. This aligns with the primary goal of prioritizing issues of fairness, accountability, transparency, and ethics (FATE) for human-AI interaction (59), specifically highlighting the importance of providing transparency to enhance users’ trust in AI-powered health applications. Our system design, including a companion app, a main commercial app, and a smart scale, resembles interconnected smart devices, offering potential applications in the health IoT field. Users’ expectations for a smooth connection between the two apps and instant synchronization of data from different sources highlight their needs for a seamlessly integrated system. More importantly, trust remains a significant concern for users transitioning from traditional devices to smart devices, emphasizing the importance of prioritizing FATE in AI-powered mHealth apps.

While prioritizing users’ specific needs and ensuring human agency in AI-powered apps, it is equally crucial to evaluate the cost-effectiveness of additional implementations. Our observation uncovered users’ legacy bias (60) from their experiences with other mHealth apps, leading them to expect our research-grade app to incorporate the comprehensive functionality and aesthetic of a commercial app. Not meeting these expectations may impact the app’s usability, but trade-offs were necessary to achieve our main design goal: effectively supporting the implementation of our team’s specific adaptive weight intervention and prioritizing the progress of our clinical trial aimed at evaluating the intervention’s effectiveness. Constantly revisiting the main research purposes of the app and involving domain expertise in the design process are essential to balance this trade-off.

Overall, the implications derived from users’ mental models emphasize the significance of understanding target users’ specific needs, applying behavior change theories, providing user autonomy, ensuring seamless integration and trust in AI-powered devices, and recognizing users’ legacy bias.

### 6.2 Reflection on our human-centered design process

Prior work (61) has demonstrated the effectiveness of including trained and seasoned experts throughout the design process of an mHealth app. By incorporating domain experts into the design process, designers can tap into specialized knowledge, gain deeper insights into the domain, and create solutions that effectively address user needs while also meeting domain-specific requirements. Our findings highlight the importance of acknowledging that user preferences may not always align with optimal design choices, particularly when target users may not possess the expertise in the specific field that the designer aims to contribute to. In our case, user preferences could have been influenced by their prior experiences with applications lacking clinical and behavioral health motivation. Therefore, while prioritizing the alignment of our design with user needs, we also consulted with our health experts to maintain adherence to the current best practices recommended in the weight maintenance field.

The iterative nature of a human-centered design process facilitated continuous improvement of our app. Through incremental small changes, we targeted specific aspects of the app, allowing us to closely identify and address user needs, gauge the impact of each update, and make necessary refinements. In our early usability study, we worked with students from our local university to gather general usability insights and make iterative improvements before progressing to testing with the target users, who are more costly and difficult to recruit. We aimed to wait until basic bugs and obvious usability flaws were fixed before going to our target user population. Although testing with a target audience is ideal, testing with students in the domain of interest (e.g., Computer Science) can still uncover high-level usability issues that are common across different user groups and provide valuable insights into the general user experience early in the design process (49). Specifically, pilot study participants appreciated the in-app notifications, the ability to access detailed information by clicking on the graphs, and the numerical data highlighting calorie differences.

It is important to note that this convenience sampling approach involves challenges to generalizability. It can be prone to high sampling bias and hence reduce representativeness (50). Specifically, since our major goal is to enable efficient research progress, we adopted Nielsen’s concept of discount usability engineering (49) by creating real-world scenarios, instead of conducting an in-the-wild testing. However, a laboratory-based study may still not fully replicate real-world stress. Furthermore, when compared to our target user group, the students we recruited were younger and were not necessarily aiming to maintain their weight after a certain amount of weight loss. These differences in demographics and objectives could potentially introduce biases. As Nielsen (49) pointed out, when testing with students, researchers should consider whether the system is also intended to be used by older users. Also, prior work has indicated that when mHealth services are perceived as personalized, younger consumers tend to be more receptive to adopting them (62). This highlights the importance of continuously reassessing our design goals and critically evaluating whether specific features align with our objectives. We mitigated potential biases in our design process by engaging health experts to conduct heuristic evaluations that follow recommendations from the weight management literature for each incremental refinement.

Overall, the reflection on our human-centered design process emphasizes the importance of involving domain experts throughout the design process because user preferences may not always align with optimal design choices. We also highlight the value of working with a non-target population, which is less difficult to recruit, in the early design phase to address obvious issues, while considering potential biases stemming from contrasting preferences between user groups.

## 7 Limitations and future work

There are several limitations to our work. First, as mentioned previously, we recruited local university students instead of the target user group for the initial usability stage. While this decision had advantages (rapidly advancing the design process), it also carried the risk of falling to a local optimum without feedback from target users. To mitigate this risk, we employed mental walkthroughs of target scenarios during the usability study and actively engaged health experts in the decision-making process for refining our design. Future work can consider involving target users at earlier stages to better tailor designs to their needs.

Second, it is important to note that we did not perform a comprehensive analysis of the conversations in our semi-structured interviews and measures of usability from questionnaire responses. However, the focus of our work was to effectively utilize an iterative human-centered design process to implement a companion app that supports the implementation and evaluation of our adaptive weight maintenance intervention, rather than developing a comprehensive mHealth system for weight maintenance for all cases. Thus, we decided to prioritize the advancement of our clinical trial for evaluating the adaptive weight intervention. Future work can conduct a comprehensive analysis of the conversations in our semi-structured interviews and incorporate measures of usability from questionnaire responses to establish generalizable design guidelines on weight management app implementation, specifically for dual app use cases.

## 8 Conclusion

This paper presents the results of a user-centered design process with iterative evaluation and incremental refinements of prototypes to develop a companion app for supporting a weight loss and weight maintenance clinical trial. Our process included initial design brainstorming with our team of HCI and health experts, a basic usability study with 17 university students, and a 2-week pilot deployment with 33 target users. Overall, our project aimed to accelerate the implementation of our clinical trial on adaptive weight maintenance interventions by leveraging existing commercial apps and developing a secondary app to meet our specific research requirements, such as instrumentation needed to collect relevant data. Our primary focus was on facilitating effortless activity, food, and weight logging by the target users. We have also explored strategies to enhance motivation, self-efficacy, self-awareness, app engagement, and adherence to health goals, drawing upon behavior change theory as a guiding framework. We demonstrated the effectiveness of utilizing an iterative human-centered design process to implement a companion app for supporting the implementation and evaluation of our adaptive weight maintenance intervention.

## Data Availability

The raw data supporting the conclusions of this article will be made available by the authors, without undue reservation.

## Footnotes

^1^Also referred to as *human-centered design*.
