# Supplementary figures and images for: MyTrack+: Human-centered design of an mHealth app to support long-term weight loss maintenance
Source: Front Digit Health. 2024 Apr 22;6:1334058. doi: 10.3389/fdgth.2024.1334058 (PMC11070543; doi:10.3389/fdgth.2024.1334058)

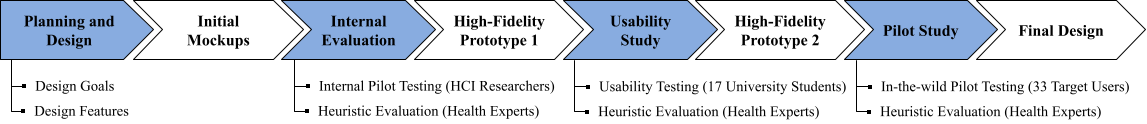

Supplement: Supplementary file 1 [file Datasheet1.zip › Data Sheet 1_v1/figures/design-process.jpg]

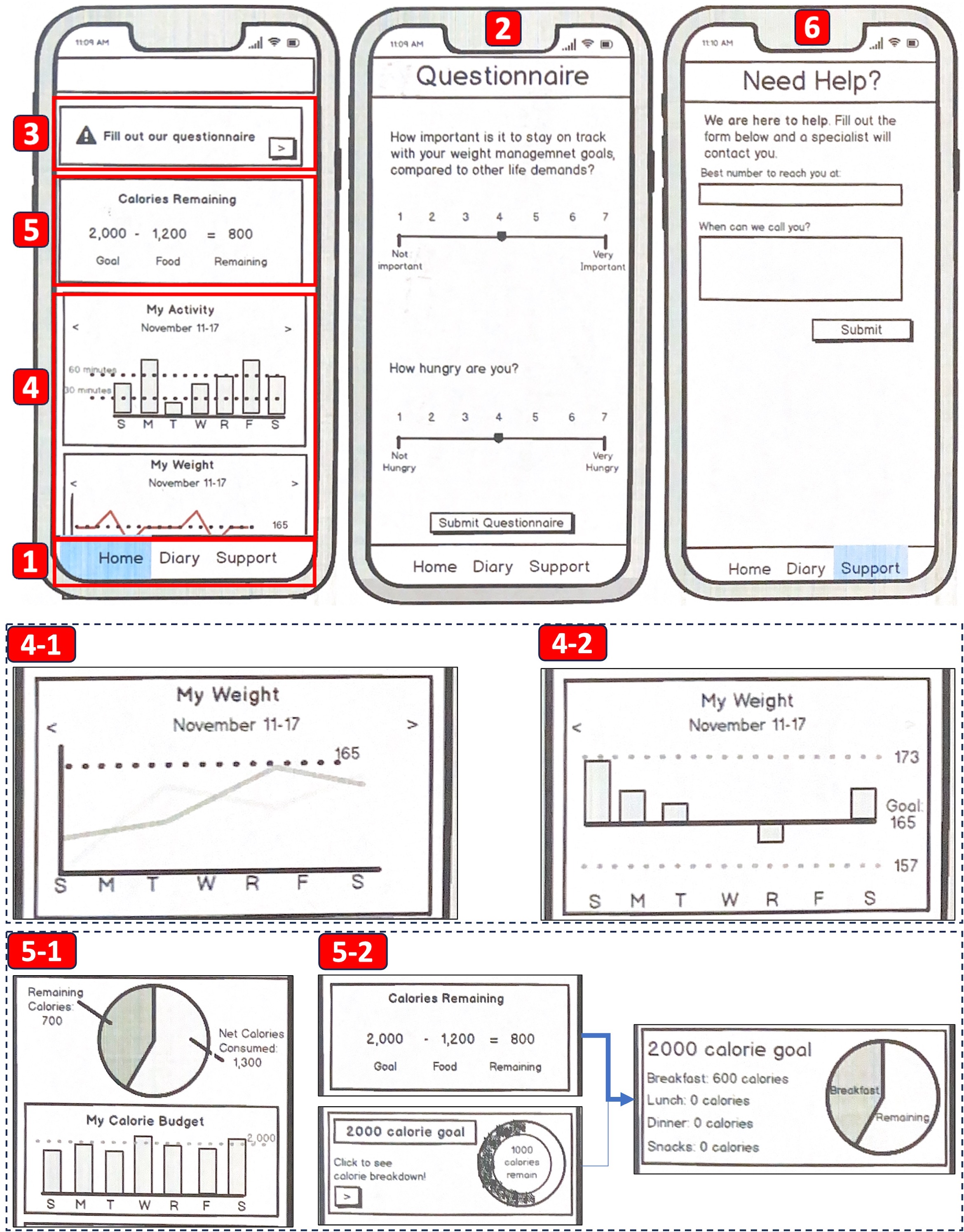

Supplement: Supplementary file 1 [file Datasheet1.zip › Data Sheet 1_v1/figures/fig1-mockup.jpg]

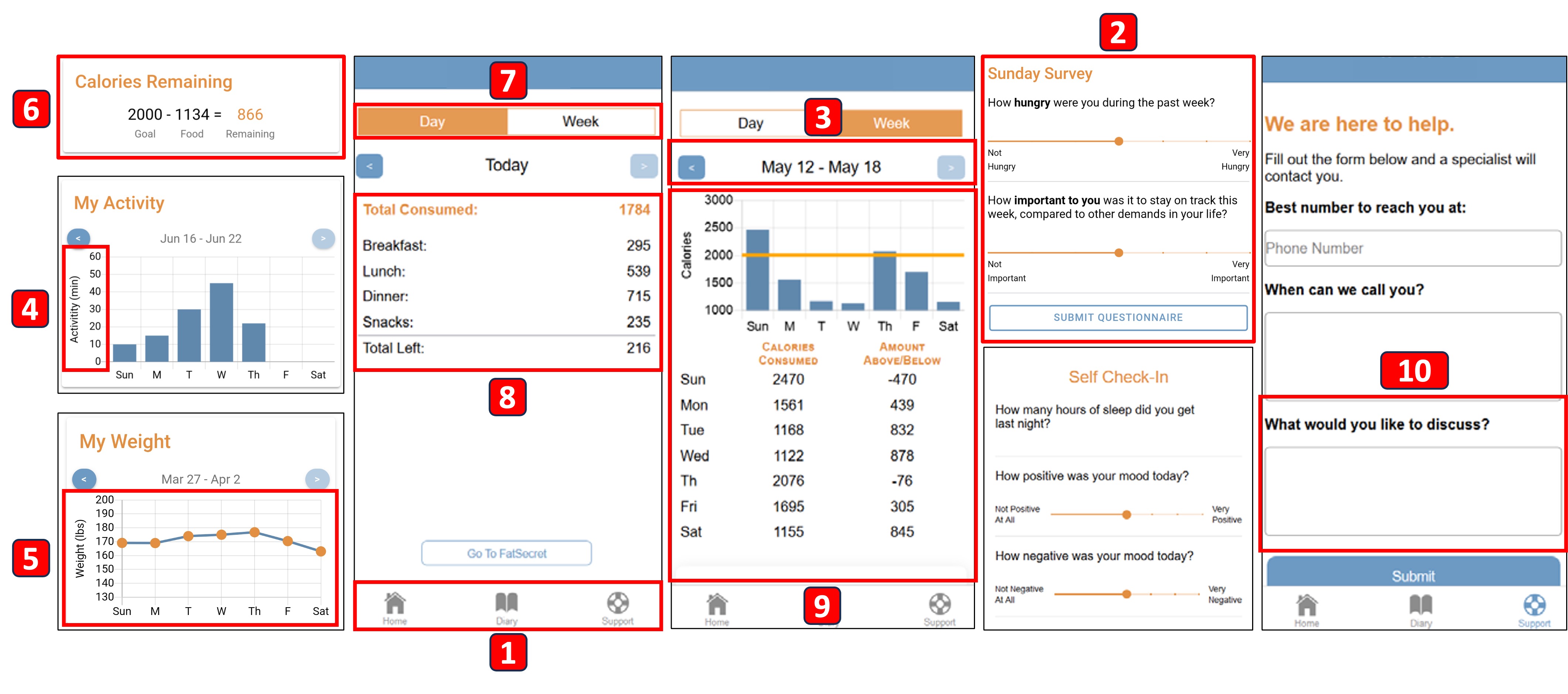

Supplement: Supplementary file 1 [file Datasheet1.zip › Data Sheet 1_v1/figures/fig2-prototype1.jpg]

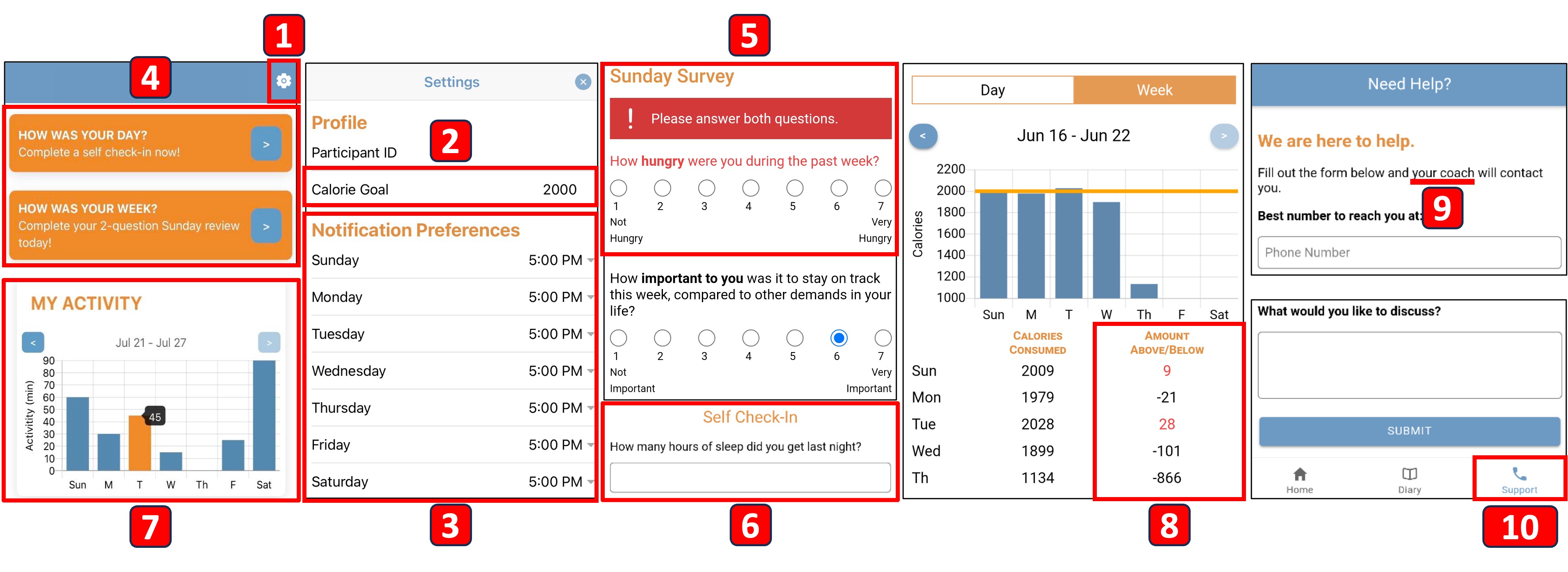

Supplement: Supplementary file 1 [file Datasheet1.zip › Data Sheet 1_v1/figures/fig3-prototype2.jpg]

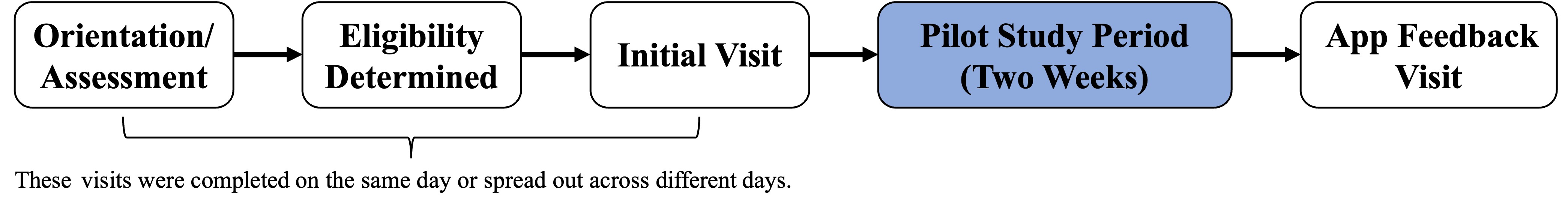

Supplement: Supplementary file 1 [file Datasheet1.zip › Data Sheet 1_v1/figures/fig4-study-procedure.jpg]

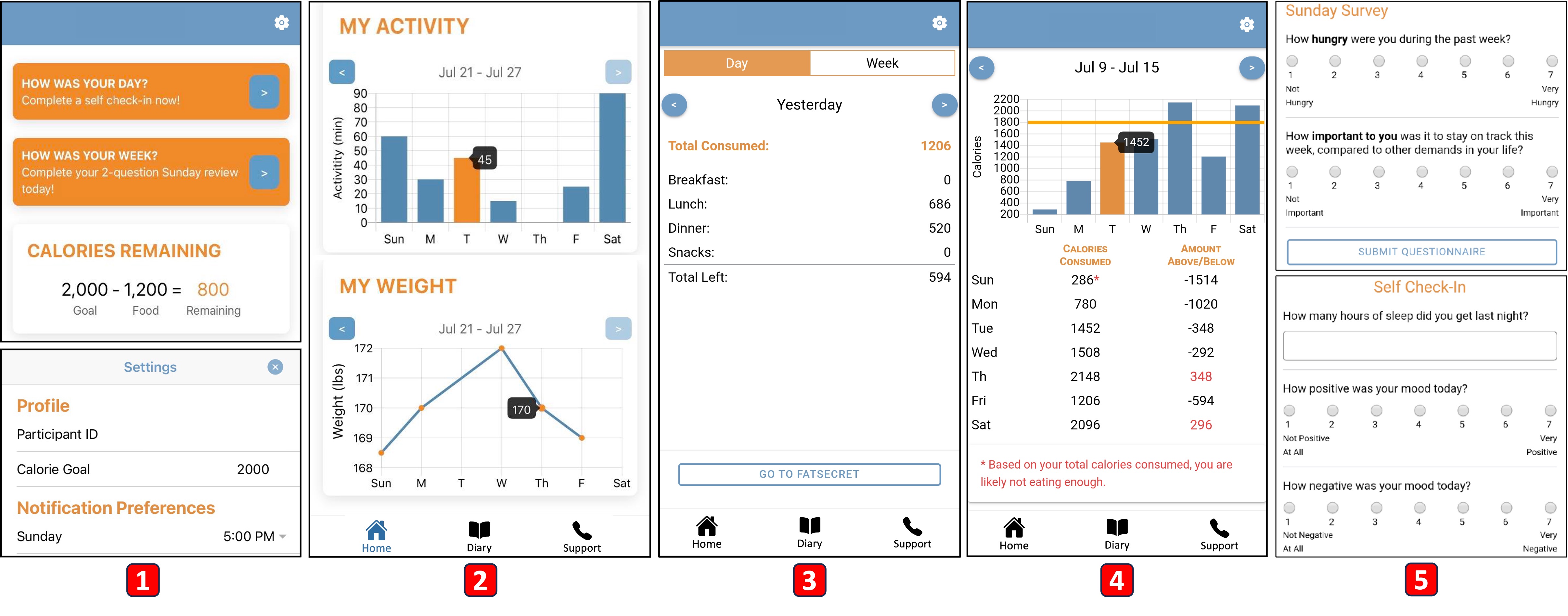

Supplement: Supplementary file 1 [file Datasheet1.zip › Data Sheet 1_v1/figures/fig5-final.jpg]
